# De novo prediction of explicit water molecule positions by a novel algorithm within the protein design software MUMBO

**DOI:** 10.1038/s41598-023-43659-w

**Published:** 2023-10-04

**Authors:** Mark Kriegel, Yves A. Muller

**Affiliations:** Division of Biotechnology, Department of Biology, Friedrich-Alexander-Universität Erlangen-Nürnberg (FAU), Erlangen, Germany

**Keywords:** Computational biology and bioinformatics, Structural biology

## Abstract

By mediating interatomic interactions, water molecules play a major role in protein–protein, protein–DNA and protein–ligand interfaces, significantly affecting affinity and specificity. This notwithstanding, explicit water molecules are usually not considered in protein design software because of high computational costs. To challenge this situation, we analyzed the binding characteristics of 60,000 waters from high resolution crystal structures and used the observed parameters to implement the prediction of water molecules in the protein design and side chain-packing software MUMBO. To reduce the complexity of the problem, we incorporated water molecules through the solvation of rotamer pairs instead of relying on solvated rotamer libraries. Our validation demonstrates the potential of our algorithm by achieving recovery rates of 67% for bridging water molecules and up to 86% for fully coordinated waters. The efficacy of our algorithm is highlighted further by the prediction of 3 different proteinligand complexes. Here, 91% of water-mediated interactions between protein and ligand are correctly predicted. These results suggest that the new algorithm could prove highly beneficial for structure-based protein design, particularly for the optimization of ligand-binding pockets or protein–protein interfaces.

## Introduction

Water is the medium in which all biochemical processes take place. Water directly influences the thermodynamics of biochemical processes by contributing to changes in enthalpy and entropy in enzyme substrate recognition, protein–protein and protein–DNA interactions as well as the interaction of therapeutics with drug targets^1^. Single water molecules often provide major atomic determinants that rule the specificity and selectivity in molecular recognition and enzymatic reactions. Their role arises from the ability of water molecules to form hydrogen bond networks that bridge between polar atoms in molecular complexes as well as from the fact that whether and where water molecules are positioned in an interaction interface depends on highly specific geometry constraints^2^. The importance of water molecules in molecular recognition processes can be appreciated for example in many protein–DNA complexes. In these complexes, direct interactions between protein side chains and nucleobases that could readily explain the high sequence specificity in these complexes often remain scarce^3, 4^. However, in many such complexes, e.g. the Trp repressor, bridging water molecules are present in the interface between binding partners and their occurrence and positioning appears to be key to the evolutionary emergence of specificity and selectivity in these complexes^3, 5^.

Recent progress in computational protein design has significantly furthered our current abilities to enable the design of proteins with novel functions. However, the ultimate goal, namely to engineer proteins/enzymes at will, still remains out of reach^6, 7^. Nonetheless, numerous examples exist, where computational protein design informed the design of proteins that bind ligands of choice. However, the ligand-binding affinities of these new binding proteins rarely match the nano and picomolar affinities observed in natural proteins. Hence, many of these computational studies need to be followed-up by directed evolution campaigns to remedy these shortcomings^7–9^. Recent advances in artificial intelligence (AI) might help to overcome these limitations. However, it would still remain desirable to improve current computational protein design algorithms that are based on atomistic structure models and energy calculations since these algorithms allow to directly probe and further our current understanding of the physico-chemical determinants that govern atomic interactions in proteins.

Although water-mediated interactions often contribute significantly to the affinity and specificity of molecular interactions, most current protein design programs do not predict the location and contribution of bridging water molecules due to high computational costs^10–16^. Here, we present a novel algorithm with significantly reduced computational costs that allows to model and include bridging water molecules in side chain-packing algorithms such as those implemented in the computer program MUMBO^16^. Starting from a given protein backbone structure, MUMBO and similar programs use side chain-packing algorithms and an energy scoring function to identify the best combination of amino acids to fulfil a specific task, such as for example the specific recognition of ligands. In contrast to other attempts, our algorithm does not use pre-solvated rotamer libraries nor does it rely on rigid models with pre-defined side-chain orientations^13, 17^. Rather, our algorithm places water molecules at physically ideal positions between pairs of rotamers. We show that this algorithm is highly efficient in predicting individual water molecules, entire meshes of water molecules and water molecules bridging between ligands and protein atoms in ligand-binding sites.

## Results

### Geometry of the water protein interactions

**Figure 1:**
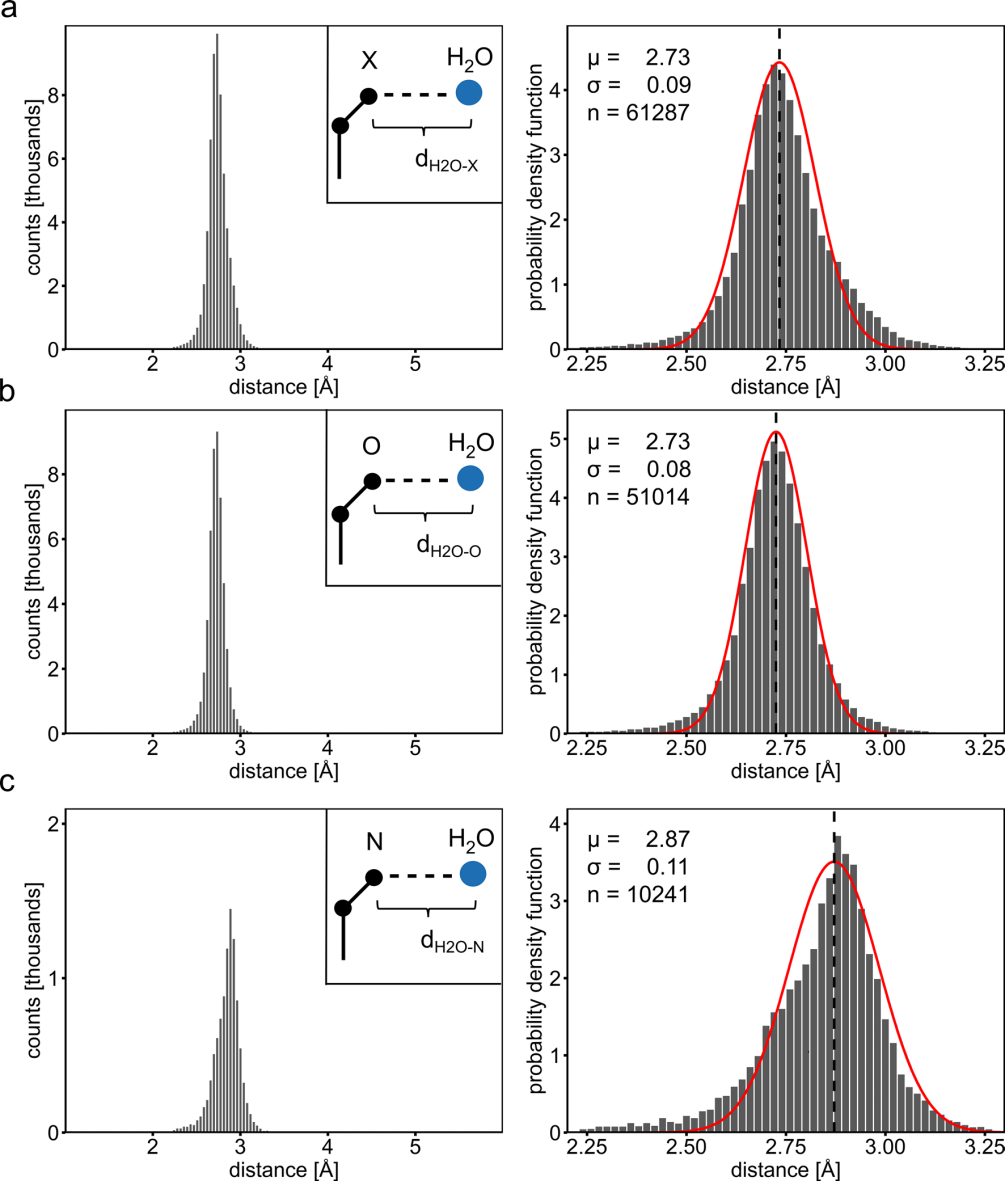
Distances between polar protein atoms and water molecules as observed in high resolution crystal structures. (**a**) Analysis of distances between water molecules and polar protein atoms in general (X) as well as individually for oxygen (**b**) and nitrogen (**c**) atoms. The number of observations (n), the fitted normal distributions (red curve), the corresponding expected value (µ, vertical line) and the standard deviation (σ) are shown.

In order to derive reliable parameters for inferring the position of water molecules interacting with protein atoms, we analysed the geometry of experimental protein-bound water molecules first. An analysis of about 60,000 crystallographically well-defined water molecules present in 590 high resolution protein crystal structures (resolution higher than 1.3 Å) deposited with the protein data bank (PDB) shows that the geometric parameters describing the water–protein interactions vary within relatively narrow ranges^18^. In the above dataset, the expected value of the fitted normal distribution for the distance (d_H2O–X_) between the water molecule (oxygen atom) and a coordinating polar protein atom is 2.73 Å with a standard deviation (σ) of only 0.09 Å (Fig. 1a). When limiting the analysis to protein oxygen atoms, an identical value is obtained (Fig. 1b), whereas a distance (d_H2O–N_) of 2.87 Å is observed for the binding of water molecules to nitrogen atoms (Fig. 1c).

**Figure 2:**
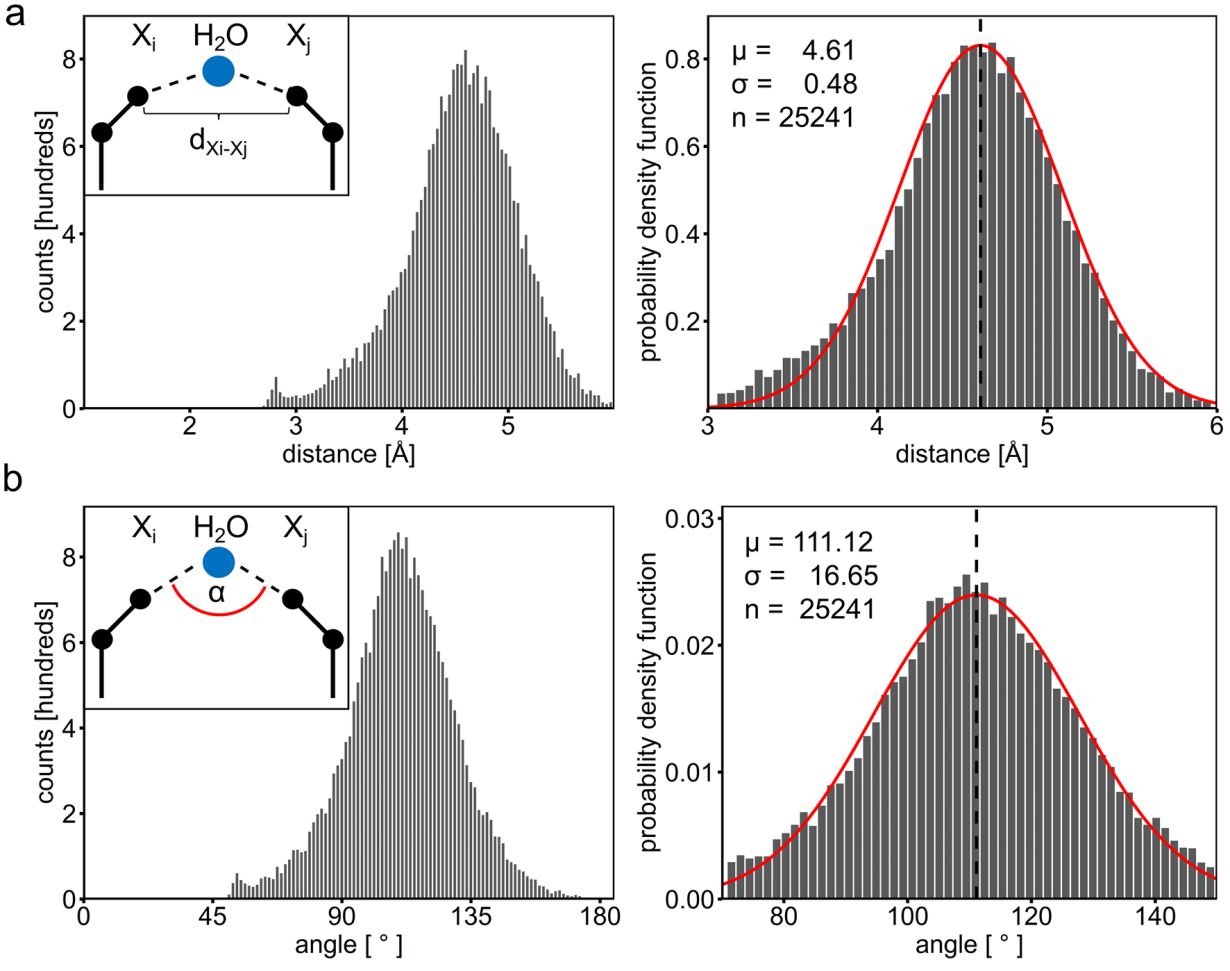
Bridging distances and angles in water-mediated interactions as observed in high resolution crystal structures. (**a**) Analysis of bridging distances (d_Xi–Xj_) between polar protein atoms. (**b**) Analysis of the bridging angles (α_Xi–O–Xj_). The number of observations (n), the fitted normal distributions (red curve), the corresponding expected value (µ, vertical line) and the standard deviation (σ) are shown.

The expected value of the distance (d_Xi–Xj_) between two polar protein atoms that interact with the same water molecule (water-bridged protein atoms) is 4.61 Å (σ = 0.48 Å) (Fig. 2a). The corresponding expected value of the angle (α_Xi–H2O–Xj_) is 111° (σ = 16.7°) (Fig. 2b). This value deviates slightly from that expected for a sp3-hybridised oxygen atom (109.5°) and hence from the expected average hydrogen-bonding angle of water molecules^19^. When assuming that the distance distribution between water-bridged protein atoms follows a Gaussian distribution, then a distance interval spanning from 4.13 to 5.09 Å (± 1σ) encompasses 68.2% of all observed distances. When enlarging the interval to 3.65 to 5.57 Å (± 2σ) then 95.4% of all observed distances fall within this bracket (Fig. 2a). The values d_H2O–O_, d_H2O–N_ and d_H2O–X_ for protein–water hydrogen bonds are in agreement with distance ranges previously observed in a study of protein–bound ligand interactions^20^.

Our analysis shows that the distributions of the d_H2O–O_, d_H2O–N_ and d_H2O–X_ distances display very sharp peaks with σ values of 0.08, 0.11 and 0.09 Å, respectively. In contrast, the angle at the bridging water molecule appears to vary more widely as reflected by a σ of 0.48 Å of the d_Xi–Xj_ distance distribution. Hence, we focused the algorithm on building water molecules at ideal d_H2O–X_ distances and allowed the angle to differ more widely from the ideal value (see below). In general, and except for nitrogen, we used the d_H2O–X_ value to predict the position of water molecules that bridge between side-chain rotamer pairs as well as between side-chains and ligands. For nitrogen atoms, the d_H2O–N_ value was applied. Furthermore, a d_Xi–Xj_ distance cut-off was used to limit the α_Xi–H2O–Xj_ angle.

### Building solvated rotamer pairs

Computer program MUMBO makes use of rotamer libraries to build amino acids and their preferred orientations at user-specified sites. It then relies on side chain-packing algorithms and an energy scoring function to identify the best combination of amino acid types and side chain conformations to derive new ligand-binding sites and novel protein–protein interaction surfaces^16, 21, 22^. In the past, attempts have been reported to extend programs similar to MUMBO to also predict the positions of water molecules as part of the design process. This was achieved by adding solvated rotamers to the rotamer libraries, thereby drastically expanding these libraries and the number of rotamers that are considered at each position^13^.

**Figure 3:**
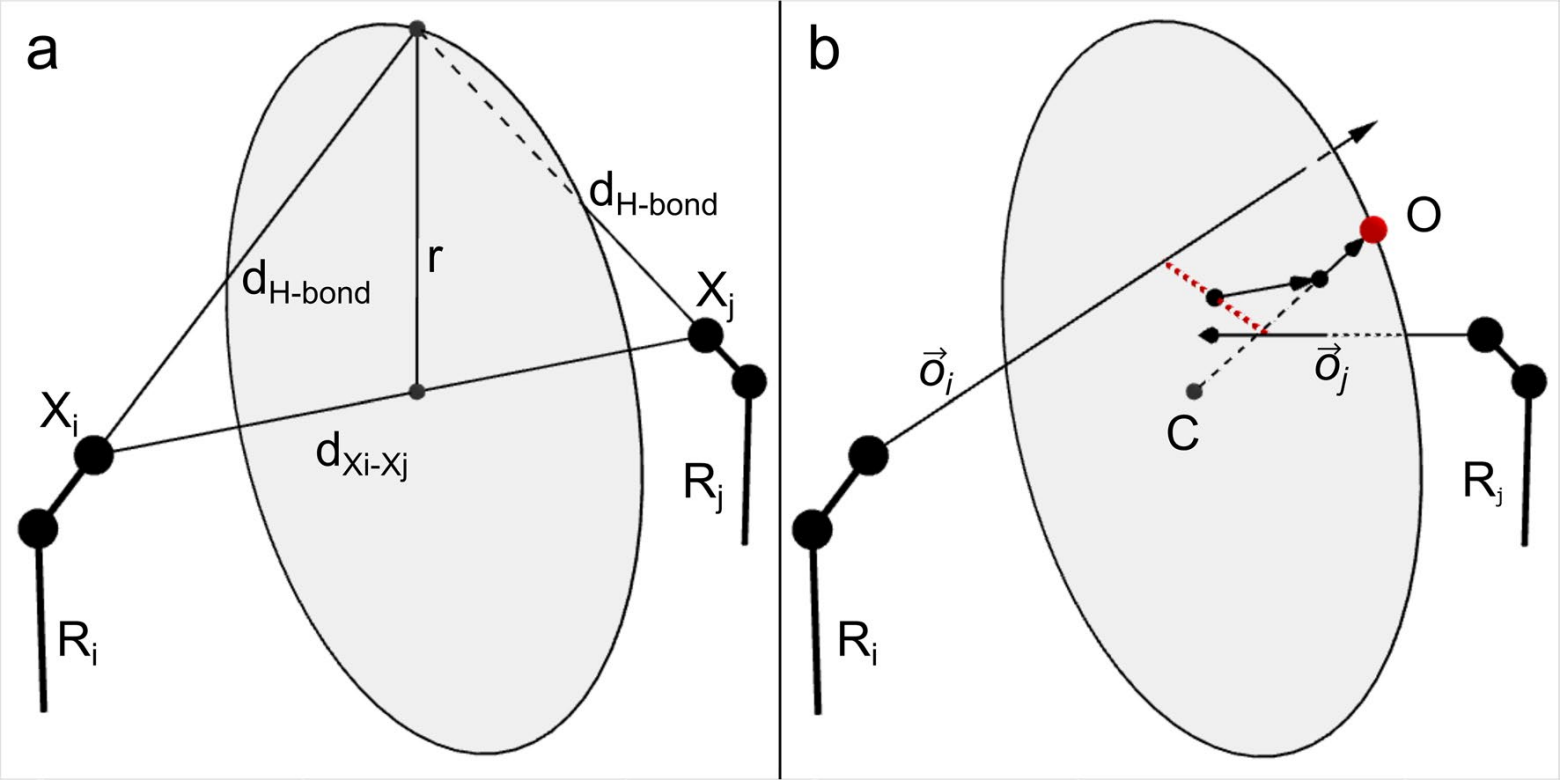
Identifying bridging water positions and building solvated rotamer pairs. (**a**) If the distance d_Xi-Xj_ between the polar atoms of the two rotamers R_i_ and R_j_ lies within defined limits, a circle with radius r perpendicular to the line between X_i_ and X_j_ and center C is generated, so that all points on the circle display the optimal H-bond distance (d_H-bond_) between the water molecule and the corresponding polar group. (**b**) The midpoint of the smallest distance between the direction/orbital vectors $\overrightarrow{{{\varvec{o}}}_{{\varvec{i}}}}$ and $\overrightarrow{{{\varvec{o}}}_{{\varvec{j}}}}$ is determined and projected onto the plane of the circle. The resulting point is then shifted to the circle perimeter along a line that interconnects this point and the center of the circle and defines the oxygen position of the newly generated bridging water molecule.

Here, we propose a novel algorithm that does not rely on expanded rotamer libraries. Instead, the algorithm adds water molecules after the rotamers have been generated. The algorithm starts out from pairs of rotamers displayed from different sites and evaluates whether a water molecule could be placed that bridges between these pairs (Fig. 3). In comparison to solvated rotamer libraries, our approach reduces the combinatorial complexity of the problem since in the first step, water molecules are not indiscriminately added via solvated rotamers but added at ideal positions between two potential interaction partners. In addition, as two of the anchor points of the water-mediated interactions are known, the number of possible water orientations (as defined by the orientation of the water-attached hydrogens) is reduced from an infinite number of possible orientations to no more than two.

The algorithm first checks whether both rotamers contain at least one hydrogen bond acceptor or donor atom and that the distance between these atoms (d_Xi–Xj_) falls within the range of distances expected for water-mediated interactions (see above, Fig. 3). The algorithm then anticipates that a putative water molecule with optimal H-bond distances lies on a circle located between the two polar groups. The radius and the center of the circle are calculated via the Heron's formula (Supplementary Fig. S1)^23^, such that the distance between the circle and the polar groups (d_H2O–Xi_) matches the optimal hydrogen bond distance of 2.73 Å (Fig. 3a). In case of nitrogen atoms a distance of 2.87 Å was used.

Subsequently, the direction/orbital vectors for optimal hydrogen bond formation are determined. For donor groups, the vector interconnecting the polar atom to the donated hydrogen is used. For hydrogen bond acceptors, the orientation of the corresponding sp2 or sp3 orbitals are used as direction vectors depending on the hybridization of the acceptor atom. If the polar group can act as both donor and acceptor, the orientation of the orbitals is calculated in relation to the position of the hydrogen atom. Finally, two points on the vectors are being determined where the distance between the two vectors becomes minimal. The midpoint between these two points is then projected onto the circle plane and shifted to the circle perimeter along a line that interconnects the projected point and the center of the circle, so that a water position with optimal H-bond distances and a minimal deviation from the optimal orbitals of the polar protein groups is obtained. The resulting point is then used as the oxygen position of the predicted water (Fig. 3b).

The positions of the two hydrogen atoms attached to the oxygen of the predicted water molecule are calculated by placing the hydrogen atoms at a distance of 0.96 Å of the water oxygen and in such a way that the angle formed between the hydrogen and oxygen atoms (α_H–O–H_) amounts to 104.45°. In case the water molecule interacts with two protein acceptor atoms, the orientation of the water molecule is optimized, such that the hydrogens point towards the acceptor atoms. Vice versa, in case of two donor atoms, the oxygen lone pairs face the donor atoms and the hydrogen atoms are oriented at an angle from the donor atoms. In case of mixed donor/acceptor atoms, two different water orientations are being generated and saved.

In a final step, the H-bond energy between the water molecule and each rotamer is calculated using the MUMBO force field^16^. Depending on a user-defined energy threshold, the water molecule is either retained or disregarded. Accepted water molecules are duplicated and a water molecule added to each of the two water-bridged rotamers. In subsequent steps, these rotamers together with unsolvated copies of the respective rotamers undergo multiple elimination steps in order to obtain a final model representing the global minimum energy conformation (GMEC)^16, 24^.

Please note that rotamer main chain atoms are included in this workflow and are also evaluated as possible hydrogen bond donors and acceptors. The same also applies to ligand atoms, alternative ligand poses and conformations. However, if a water position is predicted to bridge between an amino acid and a ligand atom, the water coordinates are added exclusively to the amino acid rotamer in the current implementation of the algorithm.

### Validation of the water prediction algorithm

**Figure 4:**
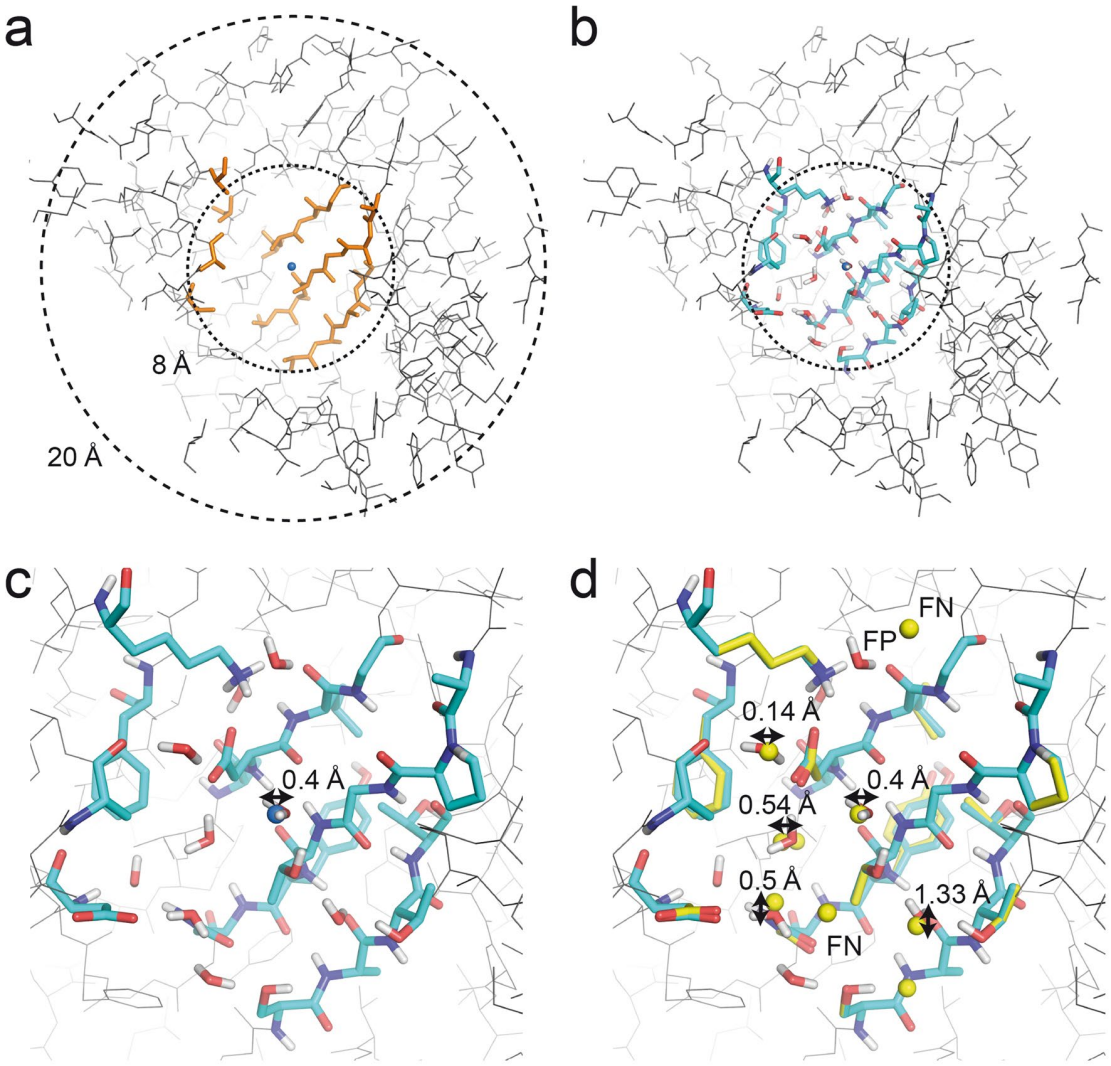
Validation of the water prediction algorithm as implemented in MUMBO. (**a**) Reference coordinate files with radii of 20 Å were edited such that all water molecules were removed and any residue with atoms located with 8 Å of the centre of the sphere truncated to alanine or glycine. (**b**) In the next step, the side chain orientations and bridging water molecules were rebuilt with programme MUMBO using the edited coordinate file and original protein sequence as input. (**c**) Close-up view of the area rebuilt in panel (**b**). (**d**) Comparison of the MUMBO-predicted side chain orientations and water positions (in cyan and red) with those observed in the crystal structure (in yellow). While in some analyses the deviation of the closest predicted water molecule to the central atom was measured (panel **a**), others analyses mapped the entire mesh of predicted water molecules to those present in the original crystal structure.

To validate the water-building algorithm, we investigated how well the algorithm is able to predict water positions, their coordination sphere as well as the orientation of the surrounding side chains in subsets with sizes of 600–1000 of the 160,000 reference coordinate files used for analysing water coordination sphere geometries (Fig. 4). In total six different calculations were performed (Table 1). Thus, in run 1 to run 3, we aimed at investigating whether water molecules with an increasing number of coordinating protein atoms can be predicted more reliably than general water molecules, including those displayed on the surface of proteins. In runs 4 and 5, we investigated how the variation of specific water-building parameters, i.e. H-bond energy and distance cut-offs, affected the results. Finally, in run 6, we addressed the question whether side chain orientations can be predicted more accurately when predicted concomitantly with water molecules.

**Table 1:** Validation of water predictions.

Run characteristics			Central water prediction characteristics		Protein side chain prediction characteristics		Water mesh prediction characteristics^b^			
ID names	Number of rotamers generated per AA^a^	Number of rebuilt spheres	Recovery rate^b^ [%]	Distance deviation (median) [Å]	RMSD of predicted spheres (median) [Å]	χ_1_ recovery rate^d^ [%]	TP^e^ [%]	Distance deviation (average) [Å]	FP^f^ [%]	FN^g^ [%]
1-bridging waters	14.8	982	66.8	0.92	1.27	86.2	48	0.81	52	64
2-semi coordinated	13.4	983	81.3	0.65	1.17	86.8	51	0.77	49	52
3-fully coordinated	13.6	611	85.6	0.59	1.15	86.8	49	0.73	51	45
4-strict distance cut-off	8.7	982	62.4	0.99	1.25	86.1	48	0.82	52	67
5-strict E_min=-4_ cut-off	6.8	982	62.3	0.98	1.25	85.9	50	0.81	50	67
6-no waters predicted	1	982	n/a^c^	n/a	1.06	88.8	n/a	n/a	n/a	n/a

Run characteristics: (1) rebuilding bridging water molecules. Water molecules are being rebuilt that are coordinated by at least two protein atoms. (2) Rebuilding semi-coordinated water molecules. Water molecules are being rebuilt if interacting with three or more protein atoms. (3) Rebuilding fully coordinated water molecules interacting with four or more protein atoms. (4) Water molecules are being built if the distance d_Xi–Xj_ satisfies the distance criterion of 4.13 < d_Xi–Xj_ < 5.09 Å (Fig. 2). All other parameters were identical to those of run (1). For comparison, in (1)–(3) and (5), this criterion was set to 3.65 < d_Xi–Xj_ < 5.57 Å. (5) In this calculation and in comparison to reference run (1), a higher and more restrictive energy cut-off of − 4 kcal/mol is applied for retaining water molecules during the water-building process. (6) Rebuilding coordinate spheres without predicting water molecules.

^a^Average number of rotamers generated at each amino acid position prior to any combinatorial rotamer elimination. Values normalized with respect to run (6). In absolute numbers, 8.9 rotamers have been generated on average at each amino acid position in run (6).

^b^Distance cut-off = 1.4 Å.

^c^Not applicable.

^d^χ_1_ deviation < 20°.

^e^True prediction (TP); percentage of predicted water molecules matching observed water molecules.

^f^False prediction (FP); percentage of predicted water molecules not matching any observed water molecules.

^g^False negative (FN); percentage of unpredicted but yet experimentally observed water molecules.

All calculations were validated by comparing the results of individual calculations with the original coordinates in the corresponding reference files. We observed that the accuracy by which MUMBO is able to reproduce the side chain orientations is very similar in all runs with median RMSD values ranging from 1.06 to 1.27 Å and χ1 recovery rates ranging from 85.9 to 88.8% (protein side chain characteristics, Table 1). Interestingly, in run number 6 in which no water positions were predicted, the lowest RMSD value (1.06 Å) and the highest χ1 recovery rate (88.8%) are observed hinting that the water prediction algorithm does not improve the accuracy by which side chain orientations are being predicted.

**Figure 5:**
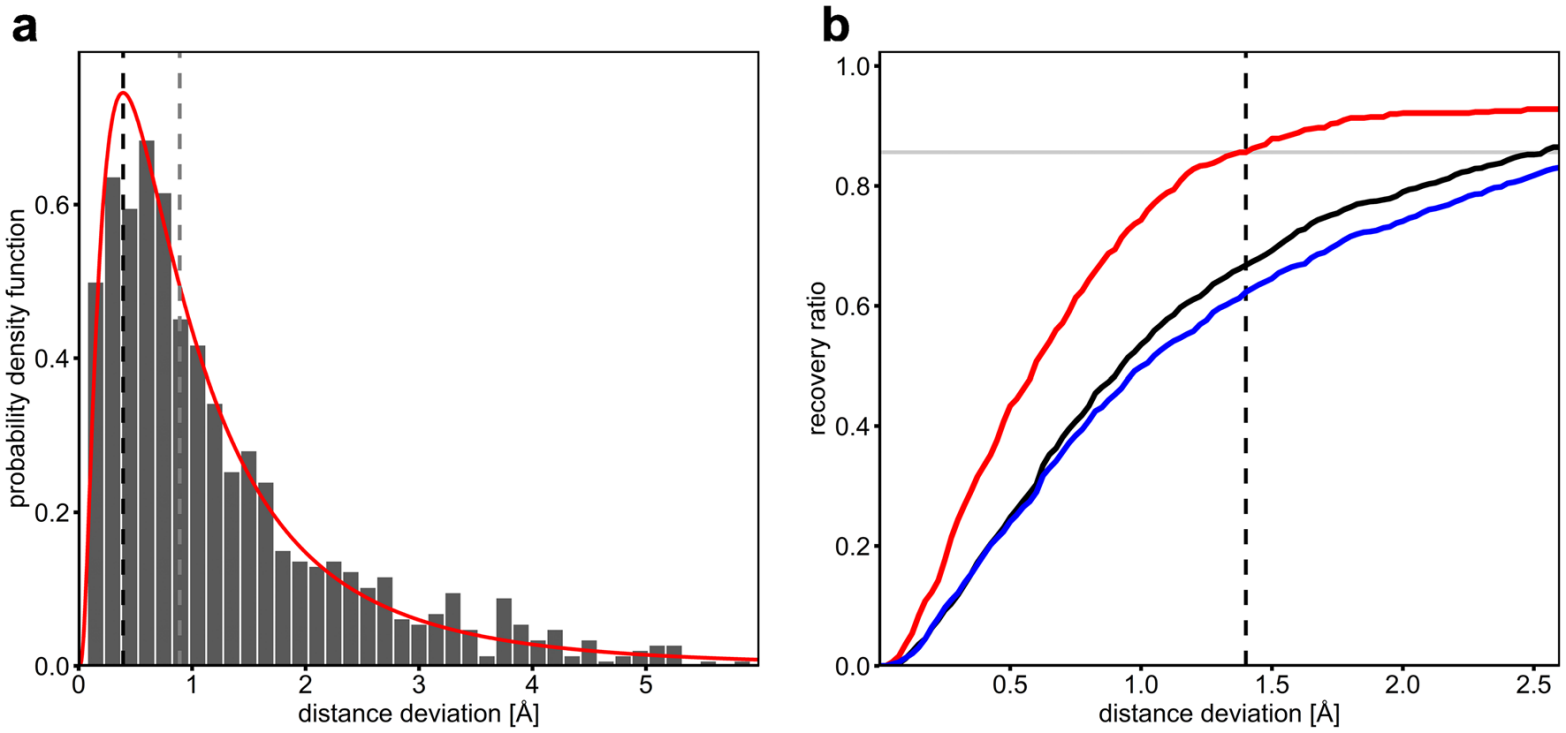
Accuracy of predicted water molecules. (**a**) Distance deviations between predicted and experimental water molecules (run 1, Table 1). The probability density function of the fitted log-normal distribution (red curve), the corresponding median of 0.89 (dotted gray line) and modus of 0.39 Å (dotted black line) are shown. (**b**) Distance deviations-dependent recovery rates of predicted versus experimental bridging water molecules. The ratio of successful predictions in general (black) as well as for the prediction of fully coordinated water molecules (red) and for water molecules with an energy cut-off of -4 kcal/mol (blue) are plotted. The recovery rate of 86% (grey line) at a distance cut-off of 1.4 Å is highlighted (dashed line) for fully coordinated water molecules.

We further investigated how reliably the algorithm is able to predict individual water molecules by comparing the position of the nearest predicted water molecule to the location of the central water molecule in individual coordinate spheres (central water prediction characteristics, Table 1). The median of the determined distance deviations between predicted and observed water position is 0.92 Å in case of bridging water molecules (water molecules coordinated by at least two protein atoms, run 1, Table 1). The distance deviations are log-normal distributed, with a mode (maximum of the probability) of the fitted log-normal distribution located at only 0.39 Å and a theoretical median of 0.89 Å (Fig. 5a). The median value significantly decreases to 0.65 and 0.59 when limiting the analysis to water molecules that are coordinated by at least three (run 2) or four protein atoms (run 3), respectively (Table 1). Such highly coordinated water atoms are often referred to as buried water molecules, and in general, the position of buried water molecules can be more easily predicted.

The recovery rate (percentage of predicted water molecules within a defined distance window) also significantly increases when comparing the prediction of less coordinated water molecules to more highly coordinated water molecules. From run 1 to run 3, the recovery rate increases from 67 to 86% when considering a distance cut-off of 1.4 Å for matching water molecules (Fig. 5b, Supplementary Table S1).

We also investigated whether more restrictive water building parameters alter water predictions accuracies. Both the application of a stricter distance cut-off for the selection of polar atom pairs to consider for water building (run 4) and the application of a stricter H-bond energy cut-off for retaining water molecules (run 5) led to only a moderate increase of the median distance deviation from 0.92 to 0.99 and 0.98 and to a slightly decreased recovery rate of 62% for both runs (Table 1, Fig. 5b). Decreasing the H-bond energy cut-off even further from − 4 to − 5 kcal/mol leads to a significant decrease in recovery rates (47.5%) and an increase of the median distance deviation to 1.42 Å (run 5.1, Supplementary Table S2). A significant advantage of more restrictive water building parameters is that they significantly reduce the number of solvated rotamer pairs that have to be processed. Hence, the subsequent rotamer elimination process is accelerated due to a reduction of the combinatorial space. Clearly, however, overly restrictive parameters can lead to a deterioration of the predictions.

In the current implementation of the algorithm, the prediction of water molecules leads to a fivefold increase in computation time (run 1) when compared to calculations with no water predictions (run 6). Encouraged by the low side chain RMSD value of 1.06 Å in run 6 in comparison to 1.27 Å in run 1 (see above), we wondered how a posterior addition of water positions would perform, i.e. applying our algorithm after a set of unique rotamers has been selected (run 6.1, Supplementary Table S2). We observe that this strategy leads to a decline of the recovery rate (from 66.8 to 46%) and an increase in the median distance deviation (from 0.92 to 1.35 Å) as seen in a comparison of the results of run 1 to those of run 6.1 (Supplementary Table S2).

### Accuracy of water mesh predictions

We used program MUMBO to rebuild entire spheres of residues and to concomitantly predict multiple water molecules located within these spheres (Table 1). The water building algorithm thus generates entire networks/meshes of water molecules. We evaluated the quality of these water mesh predictions by pairwise matching the predicted water molecules with the nearest neighbors observed in the reference coordinate files. Please note that unless otherwise stated these reference files included all crystallographically identified water molecules as well as symmetry-related copies of these water molecules (if applicable). No electron density cut-off level was applied. The pairwise matching was performed avoiding double picking. Each predicted and observed water molecule can only participate in a single match (Fig. 4d).

**Figure 6:**
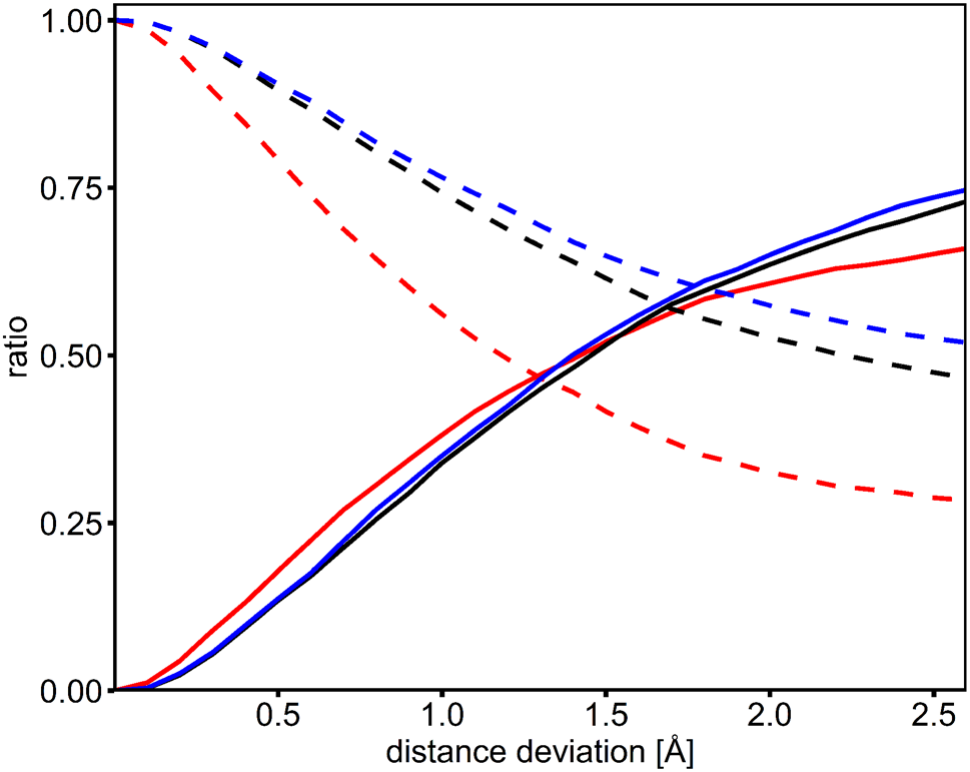
Validation of the reproduction of experimental water meshes. The ratio of true predictions (TP, solid lines) corresponding to the distance-dependent ratio of predicted water molecules matching observed water molecules as well as the false negative ratio (FN, dashed lines) corresponding to the ratio of unpredicted observed water molecules are shown. The results of the bridging water (black) data set and the full coordinated (red) water data set as well as the calculations with a 4 kcal/mol energy cut-off for the prediction of water molecules (blue) were evaluated.

We observed that in all calculations, the number of predicted water molecules is similar to the number of water molecules in the reference files. The ratios of predicted water molecules versus original water molecules varies between 0.7 and 1.2 in the different calculations (Supplementary Table S2). When considering a distance cut-off value of 1.4 Å for defining matching water pairs, between 48 and 51% of all predictions correspond to true positives (TPs, predicted water molecules with matching waters in the reference file); consequently, half of the predictions represent false positives (FPs, wrongly predicted water molecules, Table 1). The percentage of TPs increases to about 66% when increasing the distance cut-off to 2.0 Å and appears to reach a plateau at about 75% when considering distance cut-offs greater than 2.5 Å (Fig. 6). The percentage of false negatives (FNs, observed water molecules not matching any predicted water molecule) ranges from 45 (run 3) to 67% (run 4 and 5). As expected, the number of FNs is slightly higher when applying stricter geometry and energy cut-off parameters during the water building step (run 4 and 5, Table 1) since in these cases considerably less water molecules are being predicted.

Lower numbers of water molecules are also predicted when applying the water building algorithm posteriorly, namely after the selection of the best set of non-solvated rotamers. In this case, the percentage of FNs reaches 80% (run 6.1, Supplementary Table S2). However, the predicted water positions match those observed in the reference data sets quite well, thereby yielding an average TP value of 63%, which represents the highest TP value observed in all our calculations (Supplementary Table S2).

We consider a distance cut-off of 1.4 Å to be suited best for identifying matching waters and evaluating the prediction success. 1.4 Å corresponds to half the distance we previously determined for the average distance between any protein atom and attached water molecule in the reference coordinate sets (d_H2O–X_ = 2.73 Å, see above). Also, when using a distance cut-off of 1.4 Å, the average distance deviation in these water meshes between predicted and observed water molecule is 0.8 Å, a value that closely matches the median distance deviation observed while evaluating the accuracy of the prediction of single water molecules (Table 1). In summary, our results show that about 50% (percentage of TPs) of the water molecules present in water meshes are predicted correctly and with high accuracy, i.e. an average distance deviation of about 0.8 Å.

### The prediction of waters in protein–ligand interfaces

**Figure 7:**
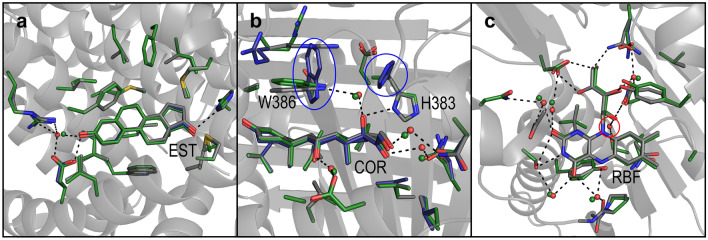
Prediction of water-mediated interactions between proteins and ligands. Comparison of the experimental structure of the ligand-binding pockets of a stabilized human estrogen receptor in complex with estradiol (**a**, EST), an alpha1-antichymotrypsin variant in complex with cortisol (**b**, COR) and an riboflavin uptake transporter A in complex with riboflavin (**c**, RBF) with the corresponding predictions (PDB entries 7NEL, 6HGF and 4IIL)^25–27^.The experimental structures (gray), the predicted structures including bridging waters (green) and the structures without predicting waters (blue) are superimposed. Predicted bridging water molecules (green spheres), as well as bridging waters in the experimental structures (red spheres) and the corresponding mediated interactions are depicted (dashed lines). Significant differences between the predicted and the experimentally observed rotamer orientation (blue frame) as well as false negative bridging waters (red frame) are highlighted.

The design of ligand-binding and protein–protein interaction sites would certainly benefit most from a correct prediction of bridging water molecules since water molecules are often key contributors to the specificity of these interactions. To demonstrate the benefits of our algorithm, we exemplarily rebuilt three different binding pockets, namely of a stabilized human estrogen receptor in complex with estrogen, an alpha1-antichymotrypsin variant in complex with cortisol and a riboflavin uptake transporter A in complex with riboflavin (Fig. 7)^25–27^. In all three cases, water molecules mediate a high proportion of polar interactions between the protein and the ligand, with 30% for estrogen binding (1 out of 3 polar interactions are water mediated, Fig. 7a), 100% for cortisol binding (4 out of 4 polar interactions, Fig. 7b) and 57% for riboflavin binding (8 out of 14 polar interactions, Fig. 7c).

Overall, the positions of 91% of the water molecules that bridge between ligand and protein atoms (10 out of 11) could be predicted correctly with a mean distance deviation of only 0.5 Å. All water molecules were placed correctly in case of the estrogen- and cortisol-binding pockets (Fig. 7a,b), while for the riboflavin interaction, 5 out of 6 waters were predicted correctly by our algorithm (Fig. 7c). In case of the estrogen- and the riboflavin-binding pocket, no significant differences in the orientation of the pocket-lining amino acids is observed when turning on and off the water-building algorithm. This is however different for the cortisol-binding pocket. When water building is turned off, the water-mediated interaction between Trp 386 and cortisol is missing. At the same time, the indole ring of Trp 386 is rotated by about 90° in comparison to the experimental structure. Triggered by this, His 383 is oriented away from the ligand losing an additional ligand hydrogen bond interaction that is present in the experimental structure (Fig. 7b). In contrast to this, the structure is reproduced very accurately when water building is enabled. This is also reflected by the RMSD values between the experimental and predicted structures with 1.84 Å for the one considering explicit waters and 2.72 Å without.

## Discussion

The prediction of explicit water positions in protein design is challenging. In particular, it is difficult to strike a balance between reasonable computational effort and prediction accuracy. Our analysis of about 60,000 crystallographic waters demonstrates, that even though surface-bound water molecules interchange rapidly in solution, the geometric parameters that describe how water molecules interact with protein atoms vary within very narrow ranges. Our algorithm takes advantage of these geometric characteristics and introduces water positions through the solvation of rotamer pairs. At the same time, the computational effort appears reasonable.

We benchmarked the success of our algorithm by investigating how well (1) individual water molecules, (2) water meshes and (3) water positions that form hydrogen-bond bridges between a protein-bound ligand and individual protein atoms are being reproduced. With regard to (1), we observed recovery rates ranging from 67% for water molecules that interact with at least two protein atoms to up to 86% for fully coordinated water molecules (distance cut-off 1.4 Å). At the same time, we consider the accuracy of the predicted positions to be quite high since the median distance deviations range from 0.59 to 0.92 Å. As expected, recovery rates and accuracy improve as the number of interacting protein atoms increases, as is the case with buried water molecules.

When considering entire water meshes, we observe that about half of the water molecules are recovered (distance cut-off 1.4 Å). The recovery rate increases to 75% using a distance cut-off of 2.5 Å. In these meshes, we observe molecules that interact with several other water molecules and only a single protein atom. This is remarkable since the algorithm places water molecules at positions where they bridge between two putative side-chain orientations. However, since these waters are then added to individual rotamers in the database, the subsequent elimination and selection process is able to yield an overall configuration in which water meshes are being formed and single protein interactions occur. Thus, the algorithm is able to predict highly solvent exposed water molecules. However, a prediction of second shell water molecules, i.e. water molecules that solely interact with other water molecules, is not possible.

A protein design task of high interest is the design of novel and highly specific ligand-binding pockets. A study on high resolution structures of protein–ligand complexes revealed that 85% of these complexes have at least one bridging water molecule, with 3 on average^28^. We have only exemplified how our algorithm is able to anticipate the location of bridging water molecules in protein–ligand complexes, and the algorithm was able to correctly predict 91% of such bridging water molecules. The knowledge gain achieved by correctly predicting water positions is particularly obvious in the structure of cortisol bound to an alpha1-antichymotrypsin variant. In the absence of water predictions, and more precisely, the omission of a single water molecule leads to a total reshaping of the binding pocket giving raise to numerous non-native interactions. Conversely, the inclusion of water predictions in the calculations correctly reproduced all protein–ligand interactions in this complex. This exemplarily underlines the knowledge that can be gained by including explicit water molecules in protein design software. We anticipate that such gains will be of significant benefit in future design studies aiming at introducing novel binding sites into proteins.

A direct comparison between the performance of the water prediction algorithm presented here and the performance of previously published algorithms is difficult. Thus, most other algorithms limit water predictions to the prediction of buried, conserved, protein–protein interface or ligand-binding site-located water molecules, whereas other algorithms do not allow for side-chain reorientations and only add water molecules to predefined protein conformations^29^. Also, different distance cut-off values ranging from 0.5 to 2.5 Å are used in the literature for the calculation of recovery rates^29–31^. Moreover, all validation parameters are highly affected by the resolution of the structures included in the reference data set, as for example at 1 Å resolution a 1.6–1.7-fold higher number of water molecules can be observed than at 2 Å resolution^32, 33^.

The most similar algorithm, Rosetta-ECO, which also simultaneously predicts rotameter orientations and water positions, was validated with a strict cut-off distance of 0.5 Å and recovery rates of 27% for two-fold and 32% for three-fold coordinated waters were obtained^31^. When using the same cut-off, our algorithm shows a similar performance and achieves recovery rates of 25% for two-fold, 37% for three-fold and 43% for fully coordinated water molecules (see also Supplementary Table S1). Apparently both algorithms master the task of simultaneously predicting the protein structure and explicit water positions similarly well. SPaDES^15^, another similar approach also based on Rosetta, reports recovery rates of 77% for waters interacting with buried interface amino acids, while we achieve 79% for general bridging water molecules (2 Å cut-off, Supplementary Table S1).

In contrast, the GridSolvate Server^17^ does not consider any flexibility in the protein structure and adds water molecules to a predefined protein conformation. The method is based on a semi explicit hydration model and uses a discrete water lattice to calculate water positions. Recovery rates of 77% are achieved for the prediction of protein–ligand interface waters (using a 1.4 Å distance cut-off), which can be further improved to about 85% when restricting the predictions to bridging waters. This extends beyond our corresponding general recovery rate of 67%. Huang et al*.*^34^ further improved such grid based methods by adding local resampling to the iterative refining step and using a neural network-based scoring function. For general water positions, they achieved water recovery rates between 31% (0.5 Å cut-off) and 64% (1.5 Å cut-off). Thus, all these algorithms appear to achieve quite similar performances with the caveat that the latter two algorithm do not allow for design flexibility, since they only add water molecules to predefined conformational states.

For future work and comparisons, we propose that a 1.4 Å distance cut-off might be best suited for validating water prediction algorithms. Protein structures are inherently flexible, and we reckon that in the pursuit of a specific protein design goal, the anticipation of a water-mediated interaction is more informative than the omission of such a water molecule because its position cannot be predicted exactly. We also reckon that the criteria we used for deriving reference data sets proved quite successful. During our validation calculations, we observed that the number of predicted water positions compares well to the number of water molecules present in the reference data sets. The latter were derived from high resolution crystal structures (resolutions better 1.3 Å), and in crystal structures, the number of modelled water molecules considerably increases as the diffraction limit increases (see above). When evaluating the water mesh predictions, we matched the predicted water molecules to the entity of water molecules observed in the crystal structures. When limiting this comparison to crystallographically well-defined water molecules, only (upon application of a 2σ electron density cut-off value), we do not observe an increase of the percentage of TPs. Instead, the percentage slightly decreases (run 1.1, Supplementary Table S2). A plausible explanation for this is that the algorithm performs similarly well at predicting strongly and less strongly bound water molecules.

The algorithm presented here provides an excellent performance in predicting water positions and considerably extends the capabilities of side chain-packing algorithms. The algorithm is also able to add water molecules posteriorly, namely after the best combination and orientations of side chains has been selected. In this case however, considerably fewer water positions are being predicted then present in reference data sets. At the same time, the predicted water positions match the observed positions quite reliably. Possibly, this feature could prove quite useful for adding water molecules for example to AI-inferred structural models such as those generated by AlphaFold2^35^.

As with any other algorithm already published, there is still room for improvements. Given the outstanding importance of individual water molecules in promoting interaction affinities and specificities, as well as the enzymatic activity of numerous enzymes, all of these efforts appear highly worthwhile.

## Methods

### Compilation of reference coordinate files

To analyse water geometries and to validate the newly implemented water prediction algorithm, we compiled a set of 160,000 reference coordinate files with each file containing the coordinates of a water molecule (i.e. oxygen atom) at its centre and the coordinates of all surrounding atoms present in the corresponding crystals structures within a radius of 20 Å (Supplementary Fig. S2). These atom coordinate spheres were generated as follows. Firstly, 1570 crystal structures were downloaded from the PDB^18^. The selected structures have been solved at resolutions better than 1.3 Å, describe proteins of molecular weights between 30 and 70 kDa and have been deposited with the PDB after 2013. Secondly, structure duplicates were eliminated as inferred from the isomorphism of the crystals used during the crystal structure determination thus yielding 590 unique crystal structures. Thirdly, a coordinate file was generated for each water molecule that displayed density levels above 2σ in the associated 2Fo–Fc electron density maps calculated with the structure factors deposited with the PDB. This file contains the selected water molecule at its centre and a list of all atoms located within 20 Å of the central water molecule. To ensure that these coordinate spheres describe the surrounding of each selected water molecule comprehensively, crystallographic symmetry and hence crystal packing were taken into account. Hence, the atom coordinates of symmetry-related molecules were added to the individual coordinate files if located within 20 Å of the central water molecule, (Supplementary Fig. S3). Taken together, each of these coordinate files contains a central water molecule (selected using a 2σ density cut-off) as well as all surrounding atoms. These include all neighbouring water molecules (with no 2σ density cut-off applied) and molecules related by crystallographic symmetry. All files were handled using C-shell scripts, the CCP4 crystallographic software package and in-house computer programs (see data availability statement)^36^.

### Analysis of water protein interaction geometries

Ideal water geometry parameters were obtained by analysing the coordination sphere of the crystallographically well-defined water molecules present at the centre of the 160,000 reference coordinate files from above. However, solely waters with a polar protein atom as closest H-bonded binding partner were further analysed. The hydrogen–bond interaction distances (d_H2O–X_) of these ~ 61,000 protein-bound water molecules were analysed independently or dependently on the nature of the interacting polar protein atom, namely for all polar atoms or separately for oxygen (n = 51,000), nitrogen (n = 10,000) and sulphur (n = 32) atoms. Due to the small number of observations, the sulphur-associated distances were not further considered in the following analysis.

The geometry of water molecules bridging between polar protein atoms was analysed next. In this context, the distances between the two water-bridged polar protein atoms was measured (d_Xi–Xj_, n = 25,200) and the protein–water–protein angle (α_Xi–O–Xj_, n = 25,200) was determined. The resulting distributions of distances and angles were analysed by fitting normal distributions to the experimental data. The determined expected values (μ) as well as the standard deviations (σ) were subsequently applied to calculate either ideal water positions or as cut-off values.

### Validation of the water-building algorithm

The water-building algorithm (see result section) was incorporated into the in-house protein design software MUMBO^16^. Program MUMBO uses as input a fixed protein backbone and uses side chain-packing algorithms and energy calculations to identify the configuration of amino acids and side chain orientations that, for a given backbone fold, yields the lowest overall energy. This configuration is often referred to as global minimum energy conformation (GMEC)^16, 24^.

The water prediction capabilities of MUMBO were evaluated by rebuilding the side chains orientations and water positions in 600–1000 entries-large subsets of the 160,000 reference coordinate files generated above. Prior to the start of each calculation, all water molecules including the central water molecule were removed, and all amino acids with any atoms within 8 Å of the sphere centre were mutated to alanine (Fig. 4). We then used MUMBO to rebuild the protein structure and water content of these 16 Å wide spheres while providing for the correct protein sequence. On average, about 20 amino acid positions were rebuilt in each calculation, and, depending on the selected strategy, 9–131 rotamers initiated at each position. The dead end elimination algorithm followed by a Monte-Carlo simulated annealing selection process was used for the combinatorial elimination of rotamers^16^.

These calculations were repeated several times while slightly varying the input parameters. In all runs, the calculations were performed with only those reference coordinate files in which the central water molecule is coordinated by at least two protein atoms (as judged by a distance cut-off of 2.99 Å). An inherent limitation of the algorithm is that only bridging water molecules can be geometrically constructed, i.e. water molecules that are attached to at least one protein and one additional protein or ligand atom. In run 2 and run 3, only those reference coordinate files were selected in which the central water molecules is coordinated by a minimum of 3 and 4 protein atoms, respectively. Here, the goal was to differentiate between buried water molecules (with an increasing number of protein-water interactions) and more surface-exposed water molecules. In run 4 and run 5, two water building parameters were varied. In run 4, water molecules were only built if the distance between the bridged polar protein atoms was between 4.13 and 5.09 Å corresponding to µ ± 1 σ of the fitted d_Xi-Xj_ Gaussian distribution, while in all other calculations, a 3.65–5.57 Å distance (µ ± 2 σ) criterion was applied. In run 5 and 5.1, water molecules were only included if, in comparison to the other calculations, an increased gain in H-bonding interaction energy was expected. In run 6, the calculations were performed without predicting any water positions in order to investigate whether or not the water prediction algorithm improves the accuracy by which side chain rotamers are being predicted. In run 6.1, water molecules were placed after the best combination of side chain orientations has been determined, thereby mimicking the prediction of water positions in structures with predefined side chain orientations.

In three additional calculations, we validated the performance of the algorithm in predicting water mediated interactions between proteins and ligands. This was achieved by rebuilding the ligand binding sites of 3 protein ligand complexes. All amino acids with atoms closer than 6 Å to any ligand atoms were substituted by alanine and the ligand position was deleted. Then the corresponding amino acids and the ligand position were rebuilt with parameters similar to run 5 but with additional fine tuning of the amino acid rotamers (χ1 values of the rotamers expanded by ± 10°). The recovery rate of the 11 water molecules that mediate interactions between protein and ligand in the three complexes (distance cut-off = 1.4 Å) as well as the root mean square deviations (RMSD) between the predicted structure and the experimental structure were evaluated.

### Evaluation of the results

To assess the accuracy by which the protein side chain orientations were predicted, we calculated the overall root mean square deviation (RMSD) and the median RMSD. The overall RMSD was calculated by summing up the interatomic distance deviations of each amino acid in each of the rebuilt coordinate spheres and then calculating the RMSD using all these individual deviations. The median RMSD was derived from the distribution of the individual overall RMSD values calculated for each rebuilt coordinate sphere. The main chain atoms and Cβ atoms were omitted in these calculations. χ1 recovery rates describe the percentage of predicted side chain orientations of which the χ1 dihedral angle deviates by less than 20° from that of the original reference structure.

In order to assess the accuracy of the water predictions, we evaluated (1) the accuracy of the prediction of individual water molecules and (2) the accuracy of predicting entire water meshes. Individual water predictions were assessed by limiting the analysis to the central water molecule in each coordinate sphere and by measuring the shortest distance between any of the predicted water molecules and the central water molecule (n = 611–983). The distance distribution was then plotted and fitted using a log normal distribution. The recovery rate was defined as the percentage of central water molecules with any of the predicted water molecules within a given distance cut-off, ranging from 0.1 to 2.5 Å. Often, a value of 1.4 Å was used. Please note that the assessment of these individual water prediction accuracies focussed on crystallographically well-defined water molecules since only water molecules displaying electron densities in excess of 2σ were selected as central water molecules in each coordination sphere.

We also assessed the accuracy by which the algorithm was able to predict entire water meshes that are present in the reference coordinate spheres. These reference coordinate spheres contained all water molecules present in the original crystal structure with no application of a 2σ cut-off (except for the central water molecule or if otherwise stated). In order to avoid bordering effects, water molecules were only matched if located within 6 Å of the central water position (12 Å wide sphere). Water molecules were considered as matches if a predicted water molecule was located within a certain match distance cut-off from a water molecule in the reference data set. Care was taken to ensure that a predicted water molecule could not match more than one reference water molecule and vice versa. Matching water molecules were considered true positives (TPs), whereas predicted water molecules that could not be matched to reference water molecules were labelled as false positives (FPs). Reference water molecules not matched by any predicted water molecules were labelled false negatives (FNs). To calculate the respective percentages of TPs, FPs and FNs, the number of TPs and FPs were divided by the total number of predicted water molecules and FNs by the total number of reference water molecules present within 6 Å of the central water position. By definition the percentage of FPs corresponds to 100—percentage of TPs. Different distance cut-offs for defining matches, ranging from 0 to 2.6 Å, were investigated and the corresponding percentages of TPs, FPs and FNs evaluated.

### Software used for data processing and representation

All structure representations were illustrated using Pymol^37^. Data processing, analysis and illustration was done using Python, R and FORTRAN 95 scripts/programs^38–40^. Geometric representations of the water building process were generated using GeoGebra^41^.

### Supplementary Information

Supplementary Information.

## Data Availability

The water-building algorithm is implemented in the latest version of computer program MUMBO. MUMBO is available as FORTRAN computer source from the software repository Gitlab (https://gitlab.com/group_muller/mumbo-software2.git). Additional computer scripts, computer programs and the data set (including 160,000 spheres and central water positions) used in the present study have been deposited with zenodo.org (https://doi.org/10.5281/zenodo.8318999).
