# Supplementary material for: De novo prediction of explicit water molecule positions by a novel algorithm within the protein design software MUMBO
Source: Sci Rep. 2023 Oct 4;13:16680. doi: 10.1038/s41598-023-43659-w (PMC10550942; doi:10.1038/s41598-023-43659-w)
Supplement: Supplementary file 1 — Supplementary Information. [file 41598_2023_43659_MOESM1_ESM.docx]

### Supplementary information

|  | Recovery rate | | |
| --- | --- | --- | --- |
| Distance cut-off | 1- Bridging waters | 2- Semi-coordinated | 3-Fully coordinated |
| [Å] | [%] | [%] | [%] |
| 0.0 | 0.0 | 0.0 | 0.0 |
| 0.1 | 1.2 | 2.5 | 3.6 |
| 0.2 | 6.3 | 9.8 | 12.3 |
| 0.3 | 11.8 | 19.0 | 24.4 |
| 0.4 | 18.8 | 27.3 | 33.4 |
| 0.5 | 24.8 | 37.3 | 43.4 |
| 0.6 | 30.3 | 45.3 | 50.7 |
| 0.7 | 38.1 | 52.6 | 57.1 |
| 0.8 | 43.3 | 59.1 | 64.3 |
| 0.9 | 48.3 | 64.2 | 69.4 |
| 1.0 | 53.5 | 68.0 | 74.3 |
| 1.1 | 57.8 | 72.7 | 78.9 |
| 1.2 | 61.1 | 76.0 | 82.8 |
| 1.3 | 64.2 | 78.4 | 84.3 |
| 1.4 | 66.8 | 81.3 | 85.6 |
| 1.5 | 69.2 | 83.4 | 87.9 |
| 1.6 | 72.5 | 84.7 | 88.9 |
| 1.7 | 74.7 | 86.0 | 89.7 |
| 1.8 | 76.5 | 87.3 | 91.3 |
| 1.9 | 77.5 | 88.4 | 91.5 |
| 2.0 | 79.0 | 89.7 | 92.1 |
| 2.1 | 80.3 | 90.5 | 92.1 |
| 2.2 | 81.9 | 90.9 | 92.1 |
| 2.3 | 83.0 | 91.9 | 92.3 |
| 2.4 | 84.3 | 92.1 | 92.5 |
| 2.5 | 85.2 | 92.6 | 92.8 |

**Supplementary Table S1.** Distance cut-off dependent recovery rate of experimental water molecules. Run characteristics: (1) Rebuilding bridging water molecules. Water molecules are being rebuilt that are coordinated by at least two protein atoms. (2) Rebuilding semi-coordinated water molecules. Water molecules are being rebuilt if interacting with three or more protein atoms. (3) Rebuilding fully coordinated water molecules interacting with four or more protein atoms.

| Run characteristics | | | | | Central water prediction characteristics | | Protein side chain prediction  characteristics | | Water mesh prediction characteristics^b^ | | | |
| --- | --- | --- | --- | --- | --- | --- | --- | --- | --- | --- | --- | --- |
| ID names | Number of rotamers generated per AA^a^ | Number of rebuilt spheres | Number of  reference  waters^b^ | Number of  predicted  waters^b^ | Recovery rate^d^ | Distance deviation  (median) | RMSD of predicted spheres  (median) | χ_1_ recovery rate^e^ | TP^f^ | Distance deviation  (average) | FP^g^ | FN^h^ |
|  |  |  |  |  | [%] | [Å] | [Å] | [%] | [%] | [Å] | [%] | [%] |
| 1-bridging waters | 14.8 | 982 | 6.1 | 4.8 | 66.8 | 0.92 | 1.27 | 86.2 | 48 | 0.81 | 52 | 64 |
| 1.1-2σ cut-off | 14.8 | 982 | 4.6 | 4.8 | 66.8 | 0.92 | 1.27 | 86.2 | 43 | 0.78 | 57 | 57 |
| 3-fully coordinated | 13.6 | 611 | 3.7 | 4.4 | 85.6 | 0.59 | 1.15 | 86.8 | 49 | 0.73 | 51 | 45 |
| 5-strict E_min=-4_ cut-off | 6.8 | 982 | 6.1 | 4.2 | 62.3 | 0.98 | 1.25 | 85.9 | 50 | 0.81 | 50 | 67 |
| 5.1-strict E_min=-5_ cut-off | 3.1 | 982 | 6.1 | 3.1 | 47.5 | 1.42 | 1.25 | 86.2 | 49 | 0.80 | 51 | 76 |
| 6-no waters predicted | 1 | 982 | n/a^c^ | n/a | n/a | n/a | 1.06 | 88.8 | n/a | n/a | n/a | n/a |
| 6.1-solvation of (6) | 1.3 | 982 | 6.1 | 2.0 | 46.0 | 1.35 | 1.06 | 88.8 | 63 | 0.74 | 37 | 80 |

**Table S2.** Validation of water predictions (extension to Table 1). Data extending beyond Table 1 are highlighted in grey. Run characteristics: (1) Rebuilding bridging water molecules. Water molecules are being rebuilt that are coordinated by at least two protein atoms. (1.1) Same as (1) but solely reference waters were considered in the mesh analysis that displayed density levels above 2σ in the 2Fo-Fc electron density maps. (3) Rebuilding fully coordinated water molecules interacting with four or more protein atoms. (5) In this calculation and in comparison to (1), a higher and more restrictive H-bond energy cut-off of -4 kcal/mol is applied for retaining water molecules during the water-building process. (5.1). The H-bond energy cut-off of (5) was further decreased to -5 kcal/mol. (6) Rebuilding coordinate spheres without predicting water molecules. (6.1) Water molecule positions are predicted and added after the selection of the best combination of side chain orientations.

^a^ average number of rotamers generated at each amino acid position prior to any combinatorial rotamer elimination. Values normalized with respect to run (6). In absolute numbers, 8.9 rotamers have been generated on average at each amino acid position in run (6).

^b^ average number of reference/predicted water molecules observed within 6 Å of the central water position.

^c^ not applicable

^d^ distance cut-off = 1.4 Å

^e^ χ_1_ deviation < 20°

^f^ true prediction (TP); percentage of predicted water molecules matching observed water molecules

^g^ false prediction (FP); percentage of predicted water molecules not matching any observed water molecules

^h^ false negative (FN); percentage of unpredicted but yet experimentally observed water molecules

| $\boldsymbol{A}_{\boldsymbol{\triangle}}\mathbf{=}\frac{\sqrt{\left( \boldsymbol{a}\mathbf{+}\boldsymbol{b}\mathbf{+}\boldsymbol{c} \right)\left( \boldsymbol{a}\mathbf{+}\boldsymbol{b}\mathbf{-}\boldsymbol{c} \right)\left( \boldsymbol{b}\mathbf{+}\boldsymbol{c}\mathbf{-}\boldsymbol{a} \right)\left( \boldsymbol{c}\mathbf{+}\boldsymbol{a}\mathbf{-}\boldsymbol{b} \right)}}{\mathbf{4}}$ | (S1) |
| --- | --- |
| $\boldsymbol{A}_{\boldsymbol{\triangle}}\mathbf{=}\frac{\mathbf{1}}{\mathbf{2}}\boldsymbol{a}\boldsymbol{h}_{\boldsymbol{a}}$ | (S2) |
| $\boldsymbol{h}_{\boldsymbol{a}}\mathbf{=}\frac{\mathbf{2}\boldsymbol{A}}{\boldsymbol{a}}\underset{\Rightarrow}{\boldsymbol{I} \boldsymbol{in} \boldsymbol{II}}\frac{\sqrt{\left( \boldsymbol{a}\mathbf{+}\boldsymbol{b}\mathbf{+}\boldsymbol{c} \right)\left( \boldsymbol{a}\mathbf{+}\boldsymbol{b}\mathbf{-}\boldsymbol{c} \right)\left( \boldsymbol{b}\mathbf{+}\boldsymbol{c}\mathbf{-}\boldsymbol{a} \right)\left( \boldsymbol{c}\mathbf{+}\boldsymbol{a}\mathbf{-}\boldsymbol{b} \right)}}{\mathbf{2}\boldsymbol{a}}$ |  |

A_△_ : area of the triangle

a, b, c : sides

h_a_ : height

### Supplementary Figure S1. Heron's formula^1^


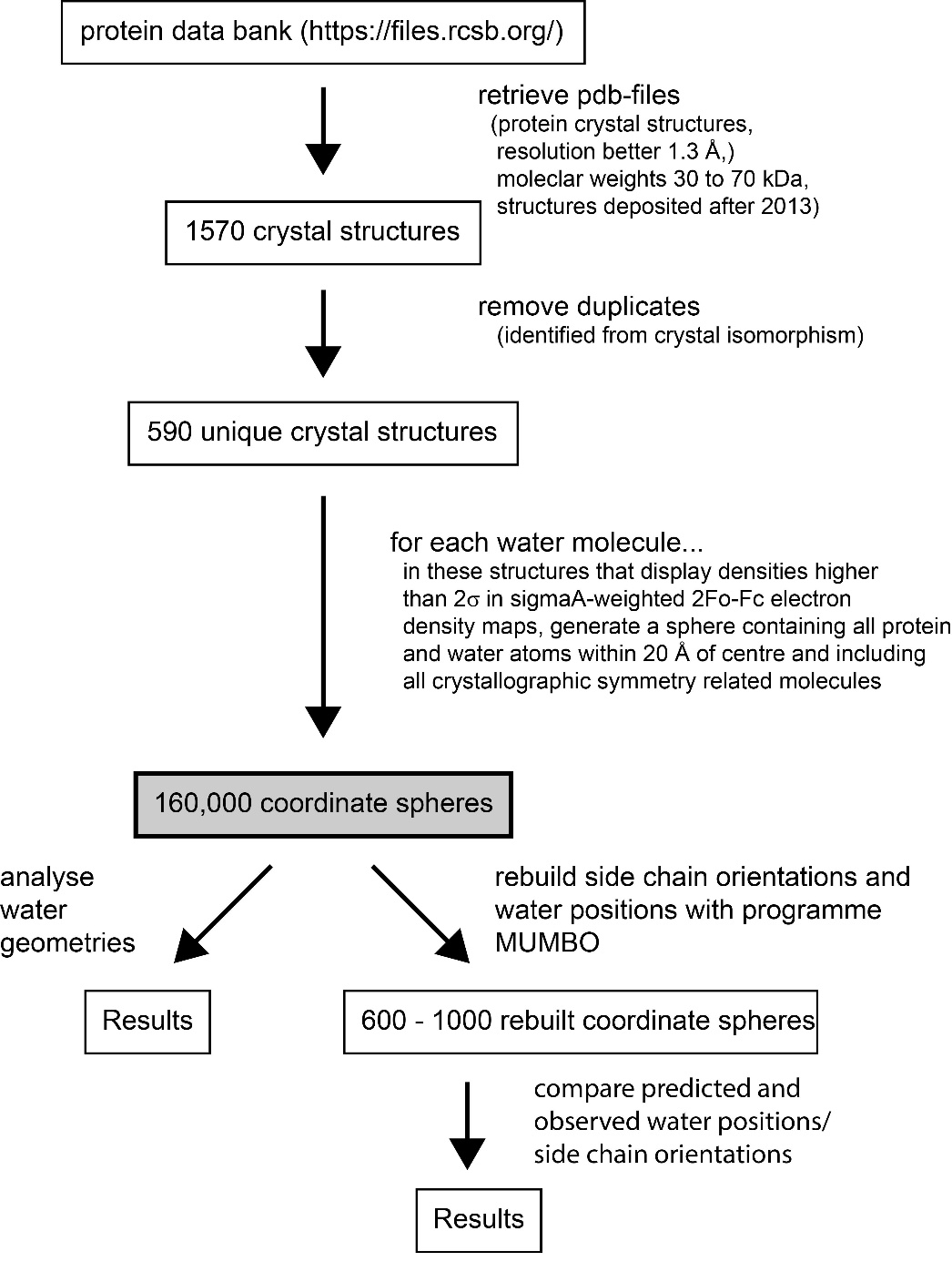


### Supplementary Figure S2. Flow scheme used for the generation of reference data sets, to analyse water geometries and to validate the water placement algorithm in computer programme MUMBO.


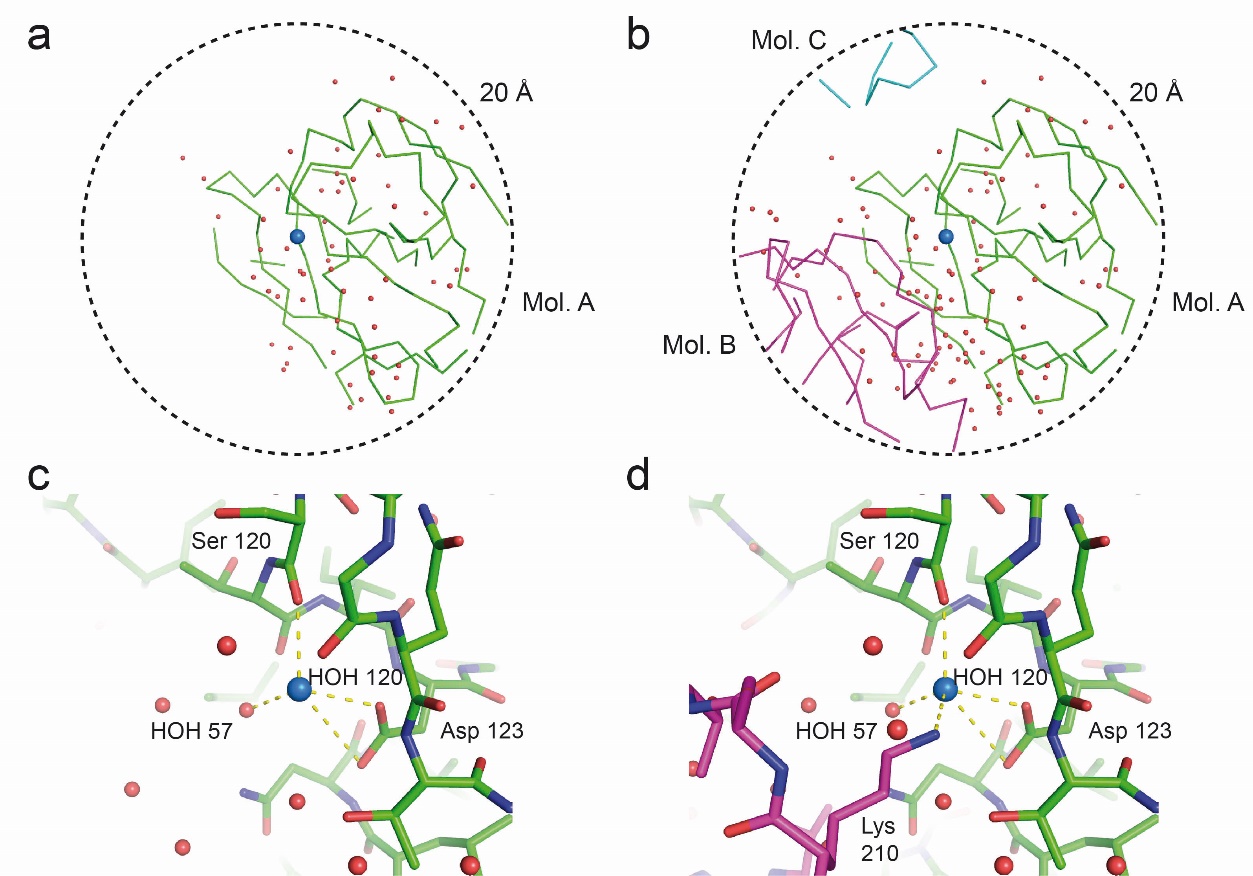


### Supplementary Figure S3. Crystallographic symmetry is applied to generate comprehensive coordinate spheres used as reference data sets. (a) All atoms within a radius of 20 Å of a central water position are selected. (b) To this selection, atoms from neighboring molecules related by crystallographic symmetry are added. (c) Close-up view of the coordination sphere of the central water molecule when omitting crystallographic symmetry. (d) Close-up view of the coordination sphere of the central water molecule when taking into account crystallographic symmetry. This example highlights that only when space group symmetry is taken into account a comprehensive description of the number and nature of the coordinating ligands as well as water-ligand distances and angles is obtained.

### Supplementary references

1 Kendig, K. Is a 2000-Year-Old Formula Still Keeping Some Secrets? *The American Mathematical Monthly* **107**, 402-415, doi:10.1080/00029890.2000.12005213 (2018).
